# The Aromatase Gene *CYP19A1*: Several Genetic and Functional Lines of Evidence Supporting a Role in Reading, Speech and Language

**DOI:** 10.1007/s10519-012-9532-3

**Published:** 2012-03-17

**Authors:** Heidi Anthoni, Lara E. Sucheston, Barbara A. Lewis, Isabel Tapia-Páez, Xiaotang Fan, Marco Zucchelli, Mikko Taipale, Catherine M. Stein, Marie-Estelle Hokkanen, Eero Castrén, Bruce F. Pennington, Shelley D. Smith, Richard K. Olson, J. Bruce Tomblin, Gerd Schulte-Körne, Markus Nöthen, Johannes Schumacher, Bertram Müller-Myhsok, Per Hoffmann, Jeffrey W. Gilger, George W. Hynd, Jaana Nopola-Hemmi, Paavo H. T. Leppanen, Heikki Lyytinen, Jacqueline Schoumans, Magnus Nordenskjöld, Jason Spencer, Davor Stanic, Wah Chin Boon, Evan Simpson, Sari Mäkelä, Jan-Åke Gustafsson, Myriam Peyrard-Janvid, Sudha Iyengar, Juha Kere

**Affiliations:** 1 Department of Medical Genetics, Biomedicum, University of Helsinki, 00014 Helsinki, Finland; 2 Neuroscience Center, University of Helsinki, 00014 Helsinki, Finland; 3 Department of Biostatistics, State University of New York at Buffalo, Buffalo, NY 14214-3000 USA; 4 Department of Psychological Sciences, Case Western Reserve University, Cleveland, OH 44106 USA; 5 Department of Biosciences and Nutrition, Karolinska Institutet, 141 83 Huddinge, Sweden; 6 Whitehead Institute for Biomedical Research, Nine Cambridge Center, Cambridge, MA 02142-1479 USA; 7 Department of Epidemiology and Biostatistics, Case Western Reserve University, Cleveland, OH 44106 USA; 8 Department of Psychology, University of Denver, Denver, CO 80208 USA; 9 Munroe Meyer Institute, University of Nebraska Medical Center, Omaha, NE 68198-5450 USA; 10 Department of Psychology, University of Colorado, Boulder, CO USA; 11 Department of Communication Sciences and Disorders, University of Iowa, Iowa City, IA 52242 USA; 12 Department of Child and Adolescent Psychiatry, Psychosomatics and Psychotherapy, Ludwig-Maximilians-University of Munich, 80336 Munich, Germany; 13 Department of Genomics, Life and Brain Centre, University of Bonn, 53127 Bonn, Germany; 14 Institute of Human Genetics, Biomedical Centre, University of Bonn, 53127 Bonn, Germany; 15 Max-Planck Institute of Psychiatry, 80804 Munich, Germany; 16 Psychological Sciences, University of California, Merced, CA 95343 USA; 17 Department of Psychology, College of Charleston, 66 George Street, Charleston, SC 29424 USA; 18 Division of Child Neurology, Department of Gynecology and Pediatrics, HUCH, University of Helsinki, 00014 Helsinki, Finland; 19 Department of Psychology, University of Jyväskylä, 40014 Jyväskylä, Finland; 20 Department of Molecular Medicine and Surgery, Karolinska Institutet at Karolinska University Hospital, 171 76 Stockholm, Sweden; 21 Howard Florey Institute, Parkville, VIC 3010 Australia; 22 Department of Anatomy and Developmental Biology, Monash University, Clayton, VIC 3800 Australia; 23 Centre for Neuroscience, University of Melbourne, Parkville, VIC 3010 Australia; 24 Prince Henry’s Institute of Medical Research, Clayton, VIC 3168 Australia; 25 Institute of Biomedicine, University of Turku, 20014 Turku, Finland; 26 Center for Nuclear Receptors and Cell Signaling, University of Houston, Houston, TX 77204-5056 USA; 27 Department of Clinical Research Center, Karolinska Institutet, 141 83 Huddinge, Sweden; 28 Science for Life Laboratory, Karolinska Institutet, 171 65 Solna, Sweden

**Keywords:** Dyslexia, SSD, SLI, Estrogen synthesis, Translocation breakpoint, Quantitative trait analysis, Categorical trait association

## Abstract

Inspired by the localization, on 15q21.2 of the *CYP19A1* gene in the linkage region of speech and language disorders, and a rare translocation in a dyslexic individual that was brought to our attention, we conducted a series of studies on the properties of *CYP19A1* as a candidate gene for dyslexia and related conditions. The aromatase enzyme is a member of the cytochrome P450 super family, and it serves several key functions: it catalyzes the conversion of androgens into estrogens; during early mammalian development it controls the differentiation of specific brain areas (e.g. local estrogen synthesis in the hippocampus regulates synaptic plasticity and axonal growth); it is involved in sexual differentiation of the brain; and in songbirds and teleost fishes, it regulates vocalization. Our results suggest that variations in *CYP19A1* are associated with dyslexia as a categorical trait and with quantitative measures of language and speech, such as reading, vocabulary, phonological processing and oral motor skills. Variations near the vicinity of its brain promoter region altered transcription factor binding, suggesting a regulatory role in *CYP19A1* expression. *CYP19A1* expression in human brain correlated with the expression of dyslexia susceptibility genes such as *DYX1C1* and *ROBO1*. Aromatase-deficient mice displayed increased cortical neuronal density and occasional cortical heterotopias, also observed in Robo1−/− mice and human dyslexic brains, respectively. An aromatase inhibitor reduced dendritic growth in cultured rat neurons. From this broad set of evidence, we propose *CYP19A1* as a candidate gene for human cognitive functions implicated in reading, speech and language.

**Electronic supplementary material:**

The online version of this article (doi:10.1007/s10519-012-9532-3) contains supplementary material, which is available to authorized users.

## Introduction

Online Mendelian inheritance in man (OMIM, www.ncbi.nlm.nih.gov/omim) documents nine loci in the human genome,* DYX1-9*, for developmental dyslexia or specific reading disability, the most common learning disorder (Scerri and Schulte-Korne 2010). Those loci and their associated genes are: *DYX1* on 15q21 (*DYX1C1*), *DYX2* on 6p22.2 (*DCDC2* and *KIAA0319*), *DYX3* on 2p16-p11 (*MRPL19* and *C2orf3*), *DYX4* on 6q11.2-q12, *DYX5* on 3p12-q13 (*ROBO1*), *DYX6* on 18p11.2, *DYX7* on 11p15.5, *DYX8* on 1p36-34 and *DYX9* on Xq27.3. Other regions and genes have also recently emerged as dyslexia candidate genes (Poelmans et al. 2009; Matsson et al. 2011).

Two different chromosomal translocations associated with developmental dyslexia have been reported in two Finnish families (Nopola-Hemmi et al. 2000). In the first family, the breakpoint was localized to 15q21 interrupting the *DYX1C1* gene at the *DYX1* locus (Taipale et al. 2003). *DYX1C1* is considered a strong dyslexia susceptibility gene and has been shown to play a role in neuronal migration, auditory processing and learning (Wang et al. 2006; Rosen et al. 2007; Threlkeld et al. 2007; Poelmans et al. 2010). Association studies of *DYX1C1* to dyslexia have been controversial; efforts to replicate the originally associated SNPs produced conflicting results suggesting that there might be another gene responsible for dyslexia in this region (Schumacher et al. 2007; Scerri and Schulte-Korne 2010). The second chromosomal translocation t(2;15)(p12;q21) segregated in a Finnish family and co-occurred in one individual with phonological awareness problems leading to severe dyslexia. The translocation maps 6–8 Mb centromeric from *DYX1C1* (Nopola-Hemmi et al. 2000), suggesting that *DYX1* might harbor another gene for dyslexia. In addition, the 15q region has also been implicated in speech and language development, specifically in speech-sound disorder (SSD), a human developmental disorder characterized by deficits in articulation and in cognitive representation of speech sounds or phonemes (Stein et al. 2006; Smith 2007; Chen et al. 2008). Also supporting a shared biology between SSD and dyslexia, is that other SSD loci co-localize with dyslexia loci, such as *DYX5* that includes the axon guidance gene, *ROBO1* (Hannula-Jouppi et al. 2005).

Early receptive and expressive language skills in early childhood have been shown to predict the later reading skills and to be linked to emergence of dyslexia in families at high risk (Torppa et al. 2010). Developmental spoken language problems are also associated with reading difficulties, for example, about 25–50% of SSD probands develop dyslexia (Raitano et al. 2004; Stein et al. 2006). Further, brain responses to auditory stimuli measured at birth, have been shown to differ between children with a familial background of dyslexia who developed dyslexia at school age, in comparison to typical readers without any familial background of dyslexia (Leppanen et al. 2010). These newborn brain responses were also associated with phonological skills before school entry and speech perception at school age. There is also evidence that specific language impairment (SLI) and dyslexia share common etiological factors that at least partly are genetically influenced (Catts et al. 2005; Newbury et al. 2011). Children with SLI have normal nonverbal intelligence but have persistent poor development in some or all of the areas of receptive and expressive grammar, phonology and vocabulary; in addition reading disorder is common among SLI children (Shriberg et al. 1999; Catts et al. 2002; Bishop and Snowling 2004). It is possible that the common etiologic link among dyslexia, SLI and SSD is in the domain of phonological processing and phonological memory (Dollaghan and Campbell 1998; Conti-Ramsden and Hesketh 2003; Pennington 2006), although each condition is recognized as a distinct developmental disorder of speech or language with its own unique characteristics as well (Catts et al. 2005; Smith 2007).

In this study, we mapped the previously uncharacterized breakpoint of the second translocation t(2;15)(p12;q21) we saw in our clinic and showed that it disrupts an area at 15q21.2, the complex promoter region of the aromatase gene, *CYP19A1*. Aromatase, or estrogen synthase, is a cytochrome P450 super family enzyme that converts C19 androgens, such as androstenedione and testosterone, into C18 estrogens, estrone and estradiol-17β, respectively. Aromatase is important in sexual differentiation and is expressed in the gonads of both sexes but also in a variety of other tissues such as the central nervous system, contributing to a local synthesis of estrogens outside of the reproductive system (Boon et al. 2010; Azcoitia et al. 2011). In the embryonic and early postnatal mammalian brain, aromatase is responsible for sexual differentiation of specific brain areas (Naftolin et al. 2001). Aromatase is also found expressed in radial glial cells of the mouse embryonic neocortex, a cell population that generates neurons during embryogenesis (Martinez-Cerdeno et al. 2006) as well as in adult radial glial cells in zebrafish, progenitor cells of the developing and adult fish brains (Diotel et al. 2010). Estrogens have important roles in brain development and neuronal differentiation by influencing cell migration, survival and death (Beyer 1999; Garcia-Segura 2008). They also have an important role in learning and memory by increasing the density of dendritic spines in hippocampal pyramidal cells and enhancing excitability and synaptic plasticity (Hao et al. 2006; Prange-Kiel and Rune 2006). Interestingly, *Cyp19a1* has an important role in the control of vocalization and behavior in songbirds and teleost fish (Forlano et al. 2006; Diotel et al. 2010).

We hypothesized that the *CYP19A1* gene, shown to be disrupted by the translocation t(2;15)(p12;q21), influences speech and language early in life, and reading at school age. Therefore, we tested the gene for association with a diagnosis of dyslexia and language-related quantitative traits (QTs) in six different samples from Finland, Germany and the USA. We also characterized several functional properties of the *CYP19A1* gene and its products, such as the correlation of its mRNA expression with other dyslexia-associated genes, in different regions of adult human brain; the binding capacity of specific transcription factors to two SNPs surrounding the brain specific promoter of the gene; and in vivo studies of the aromatase role in the growth of rat embryonic hippocampal neurons as well as in the formation of the cortex in mice. Taken together, our findings provide a broad evidence base for the role of aromatase in brain development relevant to reading, speech and language.

## Results

### The translocation breakpoint t(2;15)(p12;q21), in a dyslexic individual, disrupts the complex promoter region of the *CYP19A1* gene on 15q21.2

**Fig. 1:**
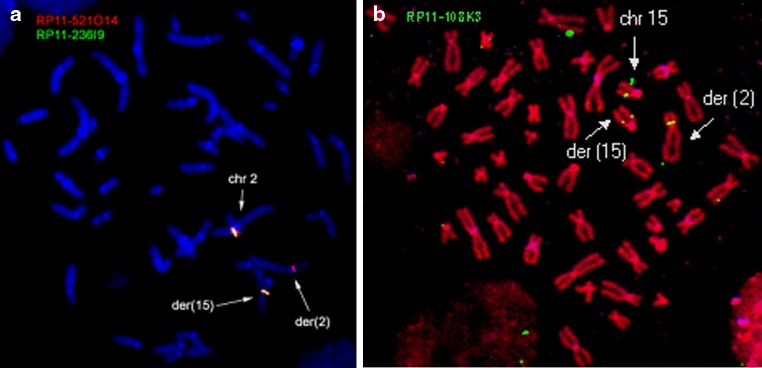
FISH detecting the t(2;15)(p12;q21) with chromosome 2- and 15-specific BAC probes, on metaphases from a dyslexic individual. **a** Chromosome 2 probe, BAC clone RP11-521O14, shows hybridization signals on chromosomes 2, der(2), and der(15) (*red*). Probe RP11-236I9, distal to the breakpoint, hybridizes only to chromosome 2 and der(15) (*green*). **b** Chromosome 15 probe, BAC clone RP11-108K3, shows hybridization signals on chromosomes 15, der(15), and der(2) (*green*)

Using fluorescence in situ hybridization (FISH) and Southern blot analysis, we refined both translocation breakpoints in the individual with t(2;15)(p12;q21) and dyslexia (Nopola-Hemmi et al. 2000). The chromosome 2 breakpoint mapped to an unremarkable region on 2q12, recognized by the bacterial artificial chromosome (BAC) RP11-521O14 and distinct from the *DYX3* locus (Fig. 1a). This 200 kb region on 2p12 is very repeat-rich and contains no known genes. Furthermore, no new genes could be identified from this region by gene prediction programs and PCR on a panel of human cDNA libraries. The gene desert stretches ~2 Mb on both sides on the breakpoint, which is ~6.5 Mb centromeric and therefore distinct from our previously reported *DYX3* locus (Anthoni et al. 2007).

**Fig. 2:**
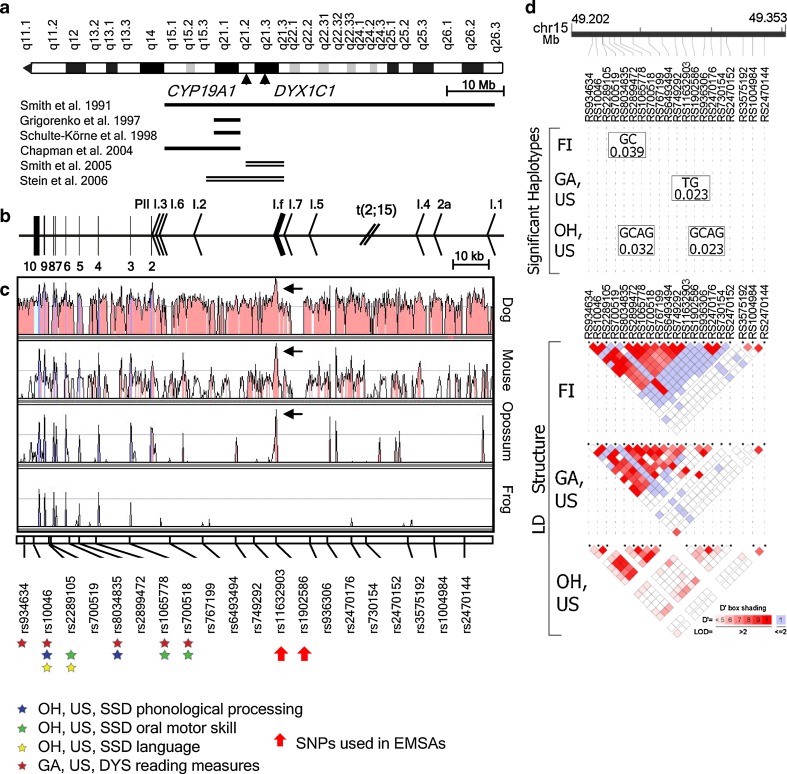
The *CYP19A1* locus on 15q21.2. **a** An overall map of chromosome 15q shows the relative positions of *CYP19A1*, *DYX1C1* and the linkage peaks in different studies of dyslexia (*solid lines*) and SSD (*double lines*). **b** *CYP19A1* gene organization, including coding exons (*vertical bars*), promoter regions (*arrowheads*), and the translocation t(2;15)(p12;q21) breakpoint (*slash*). The brain-specific exon/promoter I.f is highlighted with a *thicker arrowhead*. The gene is located on the reverse strand and therefore is drawn from right (5′) to left (3′). **c** An evolutionary comparison of the *CYP19A1* genomic sequence across four species (dog, mouse, opossum and frog) shows the highest conservation for the brain-specific exon/promoter I.f. The 20 SNPs genotyped in this study are positioned along the gene on the lowest part of the evolutionary sequence comparison. The two SNPs flanking I.f and used in EMSA experiments are indicated by *thick red arrows*. *Colored stars* under SNPs are indicating a significantly associated QT to the corresponding marker. In the OH, US, SSD cohort, association to QTs such as phonological processing, oral motor skills and language, is marked with *blue*, *green* and *yellow stars*, respectively. *Red stars* indicate association to reading measures detected in the GA, US, DYS cohort. **d** Haplotypes associated with dyslexia as a categorical trait in three of the cohorts and the respective LD structures

The chromosome 15 breakpoint mapped to 15q21.2, recognized by BAC clone RP11-108K3 (Fig. 1b), and further Southern blot analysis (data not shown) identified the exact breakpoint to the regulatory region of *CYP19A1* and more specifically, to the region between the promoter for skin, adipose tissue and fetal liver (I.4) and the promoter for fetal tissue (I.5), ~22 kb upstream from the brain-specific exon/promoter I.f (Fig. 2b).

### SNPs within *CYP19A1* are associated with the dyslexia categorical trait and with phonological phenotypes

Six cohorts of family-based material of Caucasian origin (Table 1) were genotyped for 16–20 SNPs located in the *CYP19A1* gene.

**Table 1:** Description of participants by study and sex with complete genotype and phenotype information

Cohort	Sex	Affected	Unaffected	Unknown	QTs
FI, DYS	Female	21	36	8	NA
	Male	29	27	9	NA
	Total	50	63	17	NA
GER, DYS	Female	78	27	384	350
	Male	340	21	383	297
	Total	418	48	767	647
CO, US, DYS	Female	NA	NA	NA	225
	Male	NA	NA	NA	228
	Total	NA	NA	NA	453
GA, US, DYS	Female	6	17	0	23
	Male	16	18	0	34
	Total	22	35	0	57
IA, US, SLI	Female	NA	NA	NA	240
	Male	NA	NA	NA	311
	Total	NA	NA	NA	551
OH, US, SSD	Female	135	95	5	235
	Male	133	179	3	315
	Total	268	274	8	550

#### Moderate association to dyslexia as a categorical trait

To test for association with dyslexia in the Finnish (FI, DYS), the German (GER, DYS) and the Georgia (GA, US, DYS) dyslexia cohorts, as well as the SSD cohort (OH, US, SSD), pedigree disequilibrium test (PDTPHASE) was used in order to maximize power in these family and trio materials. In the GER, DYS dataset, no haplotype was significantly associated to the dyslexia phenotype (data not shown). In the FIN, DYS dataset, there was evidence of transmission distortion to dyslexia-affected offspring for one haplotype, rs8034835-rs2899472 (GC, *p* = 0.039) (Fig. 2d), stretching from intron 4 to intron 5 of the gene (Fig. 2b, c). This haplotype overlaps with one of the significant haplotypes (rs8034835-rs2899472-rs1065778-rs700518, GCAG, *p* = 0.032) in the SSD cohort when tested for association to the dyslexia trait, as ~45% of the affected individuals from this cohort are dyslexic. The other significant haplotype in the SSD cohort, rs1902586-rs936306-rs2470176-rs2470152 (GCAG, *p* = 0.023; Fig. 2d), is located more upstream of the gene, between the translocation breakpoint and the brain specific exon/promoter I.f (Fig. 2b, c). Interestingly, in the Georgia dyslexia cohort (GA, US, DYS), the only significant haplotype, rs11632903-rs1902586 (TG, *p* = 0.023; Fig. 2d) overlaps with the significant haplotype rs1902586-rs936306-rs2470176-rs2470152 (GCAG) of the OH, SSD cohort (Fig. 2d). PDT is used only for family data and therefore could not be applied to the Colorado dyslexia (CO, US, DYS) and the SLI (IA, US, SLI) cohorts, as we lacked dyslexia status for the parents of each proband. In summary, three of the four cohorts where family material was available, do show a moderate (*p*-values significant at the 0.05 level) association between dyslexia, as a categorical trait, and a number of SNP/haplotypes from the *CYP19A1* gene. The most significant haplotype in each of the Finnish and Georgian dyslexia cohorts is fully or partially overlapping with one of the two significant haplotypes from the SSD cohort (Fig. 2d), underlining SNPs within the aromatase gene as genetic components common to dyslexia and SSD.

#### Highly significant association to language and reading quantitative traits (QTs)

Based on the role of estrogens in the development of the song circuit in male songbirds, we hypothesized that variants in *CYP19A1* might be associated with speech production and phonological processing. We thus initially focused our QTs analyses on the OH, SSD cohort. The variance component test of association was used (likelihood ratio test) because it was most suitable for QT analysis. Among our most significant results (*p*-values significant at the 0.05 level after correction for multiple testing), were SNPs associated with the rate of repetition of double syllables (oral motor skill) (rs2289105, *p* = 2 × 10^−6^; rs1065778, *p* = 4 × 10^−5^; rs700518, *p* = 1 × 10^−7^) and with the repetition of nonsense words (phonological processing) (rs10046, *p* = 5 × 10^−5^; rs8034835, *p* = 4 × 10^−5^) (Fig. 2c; Supplementary Table 6). In addition, we found association between SNPs in this same region and vocabulary (rs10046, *p* = 7 × 10^−6^; rs2289105, *p* = 3 × 10^−5^) (Fig. 2c; Supplementary Table 6) which is known to be correlated with phonological processing skills (Wise et al. 2007).

We also observed an association between some of these SNPs and reading skills measured in the GA, US, DYS cohort (Supplementary Tables 7 and 8). Among the most significant associations were those with the Woodcock reading mastery test-revised (WRMT-R) word attack subtest that measures skills in grapheme–phoneme correspondence by reading nonsense words (rs934634, *p* = 9 × 10^−5^; rs10046, *p* = 7 × 10^−4^), the WRMT-R word identification subtest that measures reading of single real words (rs80347835, *p* = 3 × 10^−4^), the Gray oral reading test-3rd edition (GORT-3) passages reading task (rs700518, *p* = 4 × 10^−4^) and the GORT-3 passage comprehension task (rs1065778, *p* = 6 × 10^−4^) (Fig. 2c; Supplementary Tables 7 and 8). We did not observe any association between QTs and variants in *CYP19A1* in the CO, US, DYS and GER, DYS cohorts, nor in the IA, US, SLI cohort. We did not have any QTs to test for association in the FI, DYS cohort. In conclusion, QT analysis of association of single SNPs showed highly significant association (*p* < 10^−4^) in the OH, SSD and the GA, DYS cohorts, for the measures of oral motor skills, vocabulary, phonological processing in the SSD cohort and reading in the DYS cohort. These associations are spread over the whole coding part of the aromatase gene, exons 2–10, approximately 30 kb in size (Fig. 2c).

### Identification of a human-specific interspecies variation in an otherwise highly conserved gene

To further characterize the breakpoint region in relation to the disrupted aromatase gene, we proceeded with an evolutionary sequence analysis of both the complex promoter and the coding region of *CYP19A1*. The sequence of the brain-specific exon/promoter I.f displayed the highest conservation across a broad range of vertebrates, using GenomeVISTA alignment (Fig. 2c), which is not surprising as aromatase has been implicated in brain development in a broad range of species (Forlano et al. 2006). We sequenced the coding exons of *CYP19A1*, as well as its brain-specific exon/promoter I.f, in four non-human primate species (chimpanzee, bonobo, gorilla and orangutan) and detected 26 single base pair variants, different in human as compared to one or more of the non-human primates tested (Supplementary Table 1). However, only four amino-acid substitutions were found, all in the orangutan and none in the other primates, suggesting that *CYP19A1* is functionally highly constrained (Supplementary Tables 1 and 2). Although we applied a likelihood ratio test to analyze the selection pressure for *CYP19A1* during primate evolution, the low information content drastically reduces the power and the estimates may not be completely reliable (Supplementary Tables 3 and 4).

**Fig. 3:**
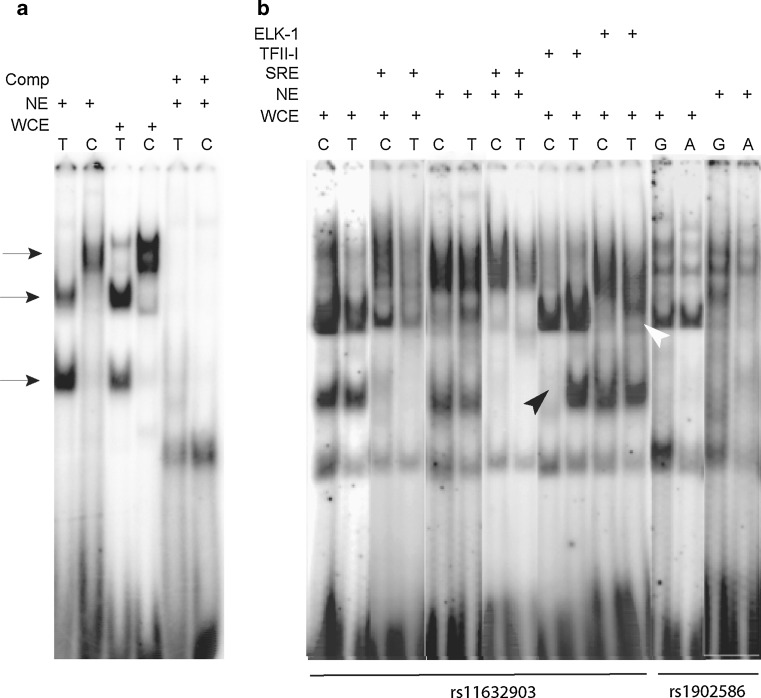
EMSA showing the effects, on transcription factor binding, of the human-specific variant from exon/promoter I.f of *CYP19A1* (**a**) as well as the effect of the two SNPs (rs11632903 and rs1902586) flanking I.f (**b**). Nuclear extracts (NE) and whole cell extracts (WCE) from the neuroblastoma cell line SH-SY5Y were used in EMSA. The specificity of all probes was confirmed by competition assays (Comp) with unlabeled probes (shown only in **a**). A “+” in the top of the EMSAs denotes the type of extracts and presence in the extracts of antibodies for supershifts or probes for competitions. **a** EMSA for the human-specific variant from exon/promoter I.f. *Arrows* show differences in retardation patterns for the human- (T) and the primate- (C) specific alleles. **b** EMSA for the two genotyped SNPs flanking I.f; *C* and *T* denote the alleles of rs11632903 and *G* and *A* of rs1902586, respectively. Predicted altered bindings of TFII-I and Elk-I to rs11632903 were verified by supershift assays (*black* and* white* *filled arrowheads* respectively) with specific antibodies, in WCE and by a consensus probe (SRE) to compete with the TFII-I binding site

Of the 26 sequence variants between human and the four non-human primates, we identified one variant that was different in humans as compared to all the other primates, including the sequences available in the public databases. This variant, a “T” in human and a “C” in all other primates, is located in the highly conserved brain specific exon/promoter I.f. Electrophoretic mobility shift assay (EMSA) experiments, using nuclear and whole-cell extracts from a human neuroblastoma cell line (SH-SY5Y), showed completely different retardation patterns for the human “T” allele as for the non-human “C” allele (Fig. 3a). In silico analysis of this sequence variant by TESS (www.cbil.upenn.edu/cgi-bin/tess/tess) and by the Genomatix SNP Inspector (www.genomatix.de) revealed a gain of transcription factor binding sites for the human-specific allele (“T”) in comparison to the non-human variant (“C”), for factors such as NEUROD1, Upstream stimulating factor ½, E-box factor, TAL-1, DEP2, c-Myc and NF-kappaE2.

### Two SNPs flanking the brain-specific exon/promoter I.f show differential binding of transcription factors

Among the SNPs genotyped in our material, rs11632903 (T/C) and rs1902586 (G/A) flank the brain-specific exon/promoter I.f of *CYP19A1* and are located ~5.8 kb downstream and ~250 bp upstream of I.f, respectively (Fig. 2c). We hypothesized that these alleles might have causative roles by affecting the binding of proteins and thus the regulation of transcription of aromatase in the brain. In silico predictions of altered transcription factor binding for both SNPs indicated differences in the number of hits as well as in the identity of the predicted binding factors. In particular, the “T” allele of rs11632903 abolished the GTF2I/TFII-I (General Transcription factor II-I) and Elk-I (ELK1, member of ETS oncogene family) binding sites that were present for the “C” allele. To verify these predicted effects, we used probes containing both alleles of these SNPs in EMSA; both SNPs showed reduced binding for the “T” in rs11632903 and the “A” in rs1902586 (Fig. 3b). TFII-I and ELK-1 bind differently with a “C” or a “T” of rs11632903 as seen by supershift assays (Fig. 3b, indicated by black and white arrowheads respectively). We also performed a competition assay with a probe from the well studied c-fos promoter, where the serum response element (SRE) binds to the TFII-I site (Kim et al. 1998). We observed a reduction of the TFII-I binding to both alleles (Fig. 3b).

### Correlation of mRNA expression in regions of adult human brain for the aromatase and six dyslexia-associated genes

**Fig. 4:**
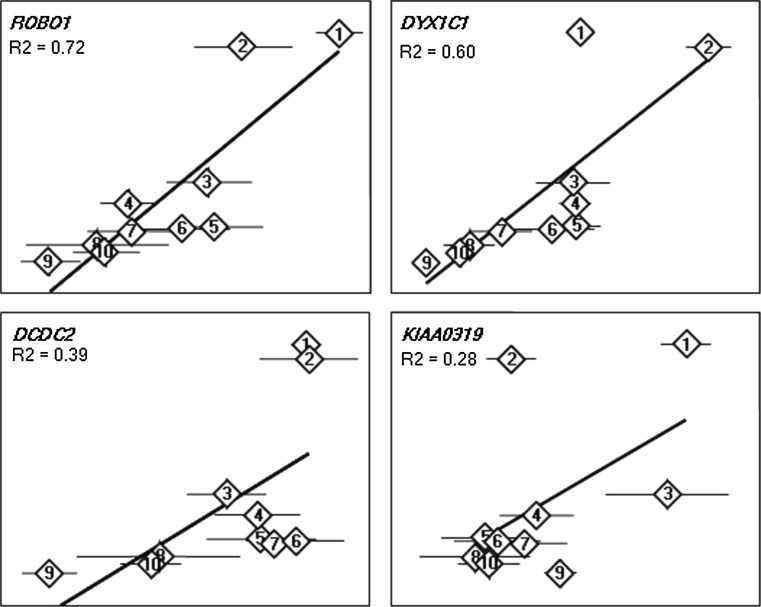
Correlation of *CYP19A1* (*y*-axis) mRNA expression to four dyslexia genes (*ROBO1*, *DYX1C1*, *DCDC2*, and *KIAA0319*; *x*-axis, respectively) in different regions of adult human brain. *X*- and *y*-axes are in arbitrary log_2_ units. For clarity, the scales are not shown. *1* thalamus; *2* hypothalamus; *3* paracentral gyrus; *4* hippocampus; *5* temporal cortex; *6* frontal cortex; *7* parietal cortex; *8* occipital cortex; *9* postcentral gyrus; *10* whole brain

As the promoter analysis suggested similar transcription factor binding sites for *CYP19A1* and the dyslexia susceptibility gene *DYX1C1*, we studied the expression levels of *CYP19A1* and the six dyslexia-associated genes reported so far. Nine sub-regions of the adult human brain were studied using quantitative real-time RT-PCR. All genes showed the highest expression in the hypothalamus or thalamus and an overall correlation across all brain regions (Fig. 4). The expression of *CYP19A1* showed strongest correlation with *ROBO1* and *DYX1C1* (*R* ^2^ = 0.72 and *R* ^2^ = 0.60, respectively), while much weaker with *DCDC2* (*R* ^2^ = 0.39) and *C2ORF3* (*R* ^2^ = 0.36) and weakest with *KIAA0319* (*R* ^2^ = 0.28) and *MRPL19* (*R* ^2^ = 0.20) (data not shown for *C2ORF3* and *MRPL19*).

### Testosterone-induced neuronal process outgrowth requires aromatase

**Fig. 5:**
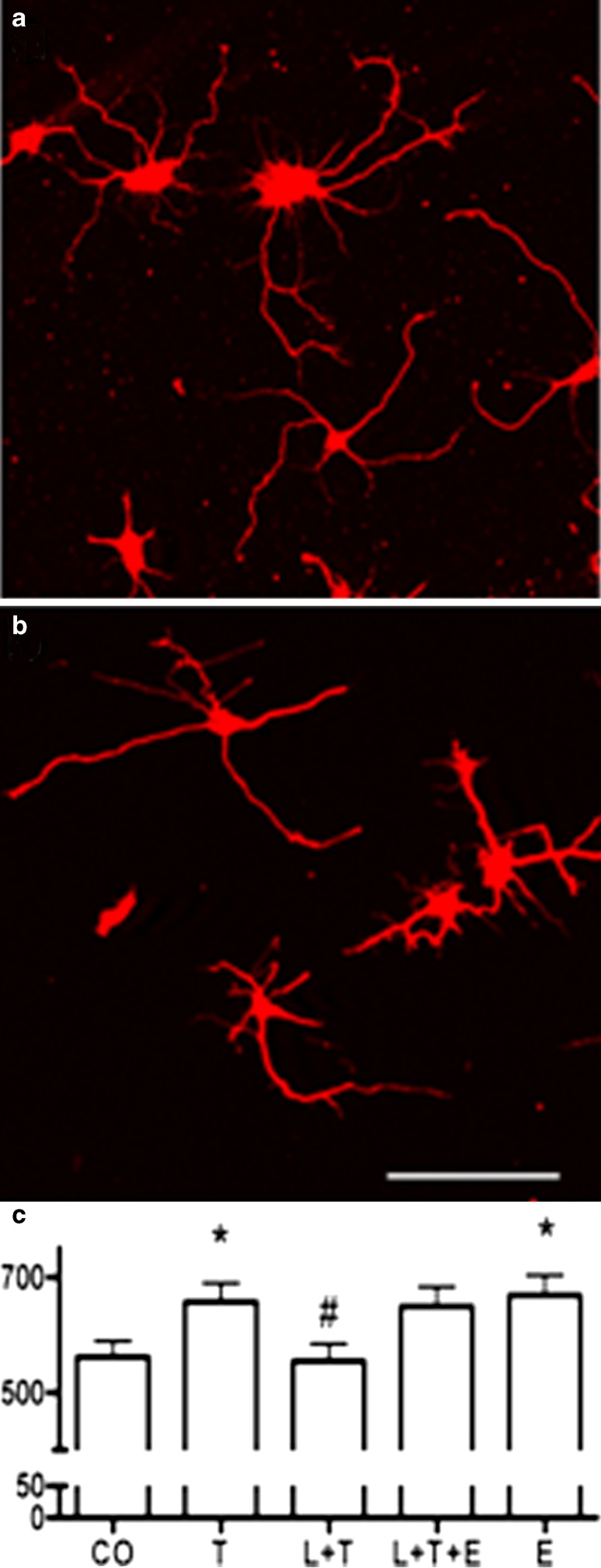
Testosterone enhances neuronal process outgrowth in an aromatase-dependent manner. E17 rat embryonic hippocampal neurons cultured for 4 days with testosterone (**a**) or with testosterone and the aromatase inhibitor letrozole (**b**), and stained with the neuronal marker TuJ1 (*red*). **c** Total neurite outgrowth in μm/neuron. The measurement shows the effects of solvent (*CO*), testosterone (*T*), letrozole (*L*), estradiol-17β (*E*). Letrozole inhibited testosterone-induced outgrowth (*L+T*), but did not inhibit the effects of estradiol-17β and testosterone together (*L+T+E*). Letrozole alone had no significant effects on neurite outgrowth. Similar effects were observed with a 3-day treatment (data not shown). *P < 0.05 against control, ^#^ *P* < 0.05 against T and L+T+E (ANOVA followed by *t*-test)

To directly study the role of aromatase function on neurite outgrowth in undifferentiated neurons, we investigated process outgrowth of rat E17 hippocampal neurons in culture. Testosterone and estradiol-17β both significantly promoted neurite outgrowth at day 4 in culture when compared to controls (Fig. 5a), also in accordance with previous reports (see review McEwen et al. 1991). The effect of testosterone was blocked by the aromatase inhibitor letrozole (Fig. 5b). However, letrozole did not block the effects of estradiol and testosterone together, indicating that aromatase-dependent conversion of testosterone to estradiol enhances neurite outgrowth in cultured hippocampal neurons (Fig. 5c).

### Aromatase knockout mice show cortical disorganization

**Fig. 6:**
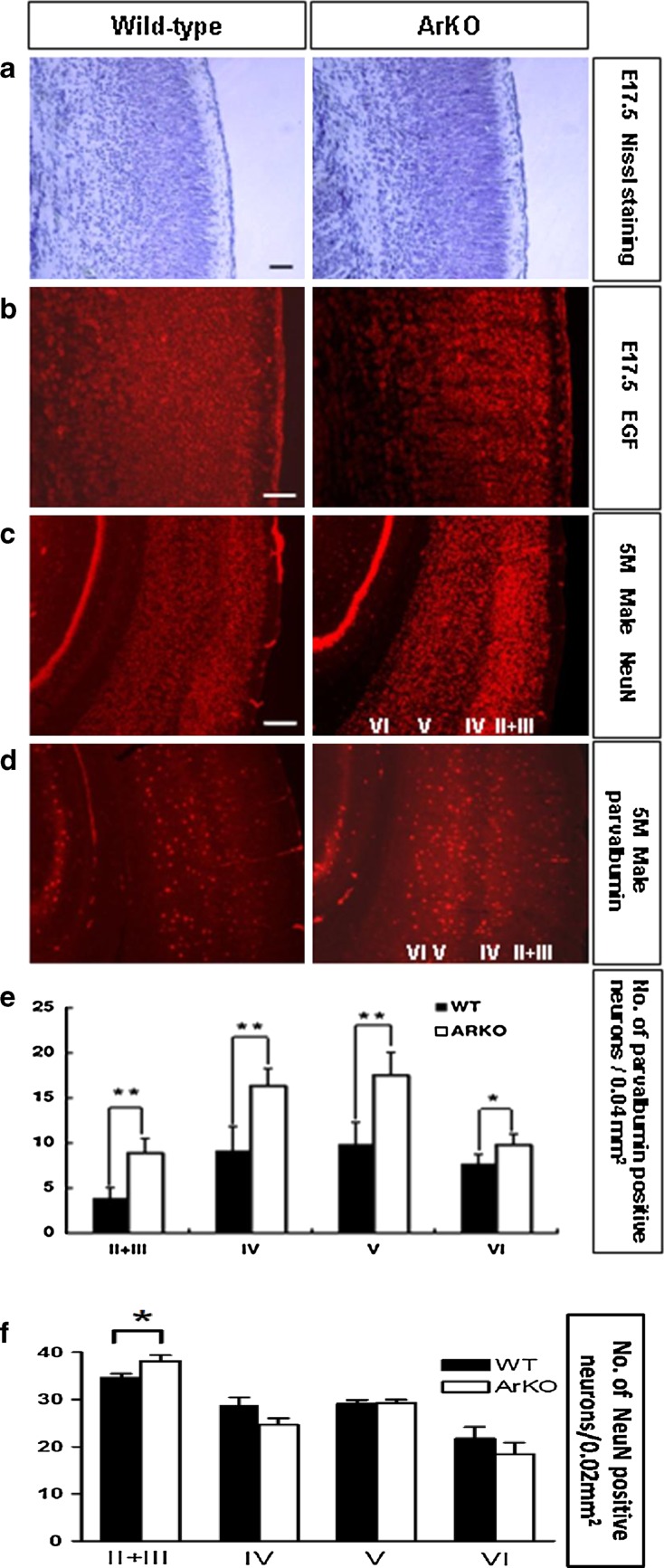
Cortical disorganization in ArKO mice as compared to WT. Increased neuronal density (**a**) and EGF signal (**b**) at E17.5 in the somatosensory cortex of ArKO mice vs. WT. Increased neuronal density (**c**) and parvalbumin-positive interneurons (**d**) in 5 months-old male ArKO mice vs. WT. (**a** cell body in *blue*; **b** EGF-positive cells in *red*; **c** neurons (NeuN-positive) in *red*; **d** parvalbumin-positive interneuron in *red*). (**e**) Mean number of parvalbumin-positive interneurons in the layers II–VI of the somatosensory cortex in 5 months-old male ARKO mice. (**f**) Mean number of NeuN positive neurons in the layers II–VI of the somatosensory cortex in 5 months-old male ArKO and WT. Student’s *t*-test: *P < 0.05; **P < 0.01. *Scale bars* **a** 50 μm; **b** 50 μm; **c** and **d** 200 μm

To study the role of *CYP19A1* in brain development, we performed a structural analysis of the brain in aromatase knockout (ArKO) mice. As shown in Fig. 6, we observed several signs of cortical disorganization in the ArKO mice as compared to wild-type (WT) controls. The neuronal density in cortical areas was significantly increased at embryonic day 17.5 (E17.5) (Fig. 6a). ArKO E17.5 mice showed also an increased signal for the epidermal growth factor (EGF) (Fig. 6b) which plays an important role in the regulation of cell growth, proliferation and differentiation. Remarkably, even in mature mice, the ArKO cortical areas had an increased neuronal density in cortical layers II/III as determined by the neuron-specific nuclear protein (NeuN) staining (Fig. 6c) and systematic cell quantification (Fig. 6f). No statistical differences between genotypes were found in other cortical layers. Moreover, an increased number of parvalbumin-positive inhibitory interneurons (Fig. 6d, e) and occasional cortical heterotopias (data not shown) were observed. The mid-sagittal areas of the anterior and hippocampal commissures (HC) as well as corpus callosum (CC), were similar in size in ArKO and WT mice (Supplementary Table 5).

## Discussion

We mapped the translocation breakpoint in a dyslexic individual carrying t(2;15)(p12;q21) (Nopola-Hemmi et al. 2000) to the complex promoter region of *CYP19A1* that encodes the enzyme aromatase. In humans, the aromatase *CYP19A1* gene stretches ~123 kb in the 15q21.2 region, with a regulatory and 5′ UTR region of 93 kb, and a coding region (exons 2–10) of ~30 kb. Its expression is regulated in a tissue- or signalling pathway-specific manner by mean of at least ten different exon 1/promoters (Bulun et al. 2004; Boon et al. 2010). The exon/promoter I.f is the brain-specific first exon/promoter located ~33 kb upstream of exon 2 and it has exactly the same length in human and mouse (139 bp) with a 94% sequence homology (Chow et al. 2009). This is by far the strongest human/mouse homology found in the different first exons/promoters of this gene, far greater than the 88.5% homology found for the ovary- and adipose tissue-specific exon/promoter 1.3.

Our evolutionary analysis of the *CYP19A1* genomic region also revealed a high level of conservation of the brain-specific exon/promoter I.f across a wide range of vertebrates, and as noted previously, it has been shown to be conserved in the songbird Zebra finch as well (Ramachandran et al. 1999). Interestingly, we identified a human-specific variation within this highly conserved brain-specific exon I.f, when comparing human and non-human primates. This variant showed different patterns of protein binding in EMSA experiments, and in silico analysis predicted differential binding of factors such as NEUROD1, Upstream stimulating factor ½, E-box factor, TAL-1, DEP2, c-Myc and NF-kappaE2. Interestingly, NEUROD1 is involved in neurogenesis, the Scl/TAL-1 transcription factor has been shown to play a key role in neuronal development (Ogilvy et al. 2007) and in Drosophila, DEP2 is binding to a steroid hormone receptor like protein (Ayer and Benyajati 1992). Further experimental studies will be needed in order to identify the true factor(s) binding to this site and regulating *CYP19A1* expression in the human brain.

The fact that the two SNPs, rs11632903 and rs1902586, that flank exon/promoter I.f, are moderately associated with dyslexia as a categorical trait in the GA, DYS cohort as a 2-marker haplotype, suggests that this very specific exon/promoter I.f might have a role in the risk for development of dyslexia. Moreover, these two variants also showed allele-specific differential binding for transcription factors such as TFII-I and Elk-1. TFII-I downregulates estrogen-responsive genes through interaction with estrogen receptor α (ERα) (Ogura et al. 2006). Elk-1 has been shown to have a role in learning and memory in rats (Cammarota et al. 2000). Interestingly, both of these factors also show altered binding for dyslexia-associated alleles at the 5′ UTR of the *DYX1C1* gene (Tapia-Paez et al. 2008), one of the main loci (*DYX1*) implicated in dyslexia.

Our results from the QTs analysis of speech and reading measures with regard to SNPs covering the *CYP19A1* gene and its entire promoter region, gave highly significant association in two (OH, US, SSD and GA, US, DYS) of the five cohorts for which QT measures were available. Specifically, we detected associations in the GA, US, DYS cohort for reading and in the OH, US, SSD cohort for measures of speech (e.g., oral motor skills, vocabulary and phonological processing). These findings support the possibility of a common mechanism for dyslexia and SSD, and the aromatase gene is, to our knowledge, the first gene which has shown association with cognitive skills related to both phenotypes. The fact that we did not observe any significant associations in the other three cohorts (GER, DYS; CO, US, DYS; IA, US, SLI) is not unusual for an association study of complex traits such as dyslexia, SLI or SSD. The cohorts from our study originate from different geographical locations, and even if these populations are primarily Caucasians, we cannot exclude the possibility that they have different genetic lineages. Therefore, the association of the aromatase gene to the reading or language-related measures we tested may not be uniform across the cohorts due to differential linkage disequilibrium (LD) between a causal variant and the genotyped SNPs, or to the actual effect size of the gene varying between cohorts (Vieland 2001). Moreover, a rare variant might be especially difficult to detect, even in cohorts drawn from a single larger population.

Aromatase is responsible for the irreversible conversion of androgens into estrogens in the developing and mature brain. Aromatase and estrogens, which can be locally produced in several tissues other than the gonads including the central nervous system, have been shown to be essential for the development of the mammalian brain, with several important roles in processes such as neuronal proliferation and migration, dendritic branching, brain plasticity and apoptosis (Beyer 1999; Forlano et al. 2006; Garcia-Segura 2008; Boon et al. 2010; Azcoitia et al. 2011). In mice of both sexes, aromatase expression level is much higher in the developing brain than in adults (Karolczak et al. 1998; Bakker et al. 2004), at a time point when the neural architecture of the brain is being determined. Our results on neuronal cultures give further support to a central role for the local aromatase activity and estrogen production in brain development (Saldanha et al. 2009). We demonstrate here that, in embryonic rat hippocampal cultures, the neurite outgrowth induced by testosterone is solely aromatase-dependant. Human aromatase deficiency is a very rare phenomenon and, to date, only 15 male and female cases have been reported (Jones et al. 2007; Lanfranco et al. 2008). However, these reports lack any description of the patient’s cognitive functions. As rodent ArKO models are available, we performed a detailed analysis of brain morphology in ArKO mice. Several signs of cortical disorganization were observed, including increased neuronal density in cortical areas and occasional cortical heterotopias. These structural abnormalities have been previously observed in the post-mortem brain of dyslexic individuals (Galaburda et al. 1985) and have also been documented in rat brains where *Dyx1c1* has been knocked-down by RNA interference (RNAi) (Rosen et al. 2007). Interestingly, similar to the ArKO mice, cortical disorganization, characterized by increased neuronal density was also observed in the brain of mice carrying a knockout of *Robo1*, another of the dyslexia-susceptibility genes (*DYX5* locus) (Andrews et al. 2006). In addition, RNAi downregulation of the dyslexia-susceptibility genes *Dcdc2*, *Kiaa0319* and *Dyx1c1* in rat embryos also affects neuronal migration to cortex (Meng et al. 2005; Paracchini et al. 2006; Wang et al. 2006). Our finding that aromatase may be necessary for neurite outgrowth in embryonic rat hippocampal cultures and for the correct structure of the cortex in mice brain, is another line of evidence supporting the importance of the *CYP19A1* gene/product in brain development. When we consider this finding alongside with findings regarding the phenotypes observed in rodents where dyslexia genes were knocked-down and the anatomical findings from human dyslexic brains, it suggests that CYP19A1 may be a significant factor in the development of the brain in areas relevant to the ability to learn and use written and spoken language.

## Methods

### Ethics statement

In this study, all research involving human participants has been properly approved by respective ethical boards from each university involved. Written consent, or assent in case of children, was obtained from all participants. The study of the Finnish dyslexia cohort was approved by Finnish ethical committees in Helsinki and Jyväskylä, Finland, as well as by the Karolinska Institutet, Stockholm, Sweden. The study of the German dyslexia cohort, recruited from the Departments of Child and Adolescent Psychiatry and Psychotherapy at the Universities of Marburg and Würzburg, was approved by the respective ethics committees. The research involving the participants in the Colorado dyslexia cohort was approved by the Institutional Review Boards of the University of Colorado, Boulder, and the University of Nebraska Medical Center. Informed consent or assent was obtained from participating adults and children respectively. For the study of the Georgia dyslexia cohort, human subject procedural approval was provided by the Institutional Review Boards of the Universities of Georgia and Purdue, USA. The study of the Iowa SLI cohort was approved by the University of Iowa Internal Review Board prior to initiation. The parents of all children were informed and provided written consent for behavioral assessment and collection of DNA from their children. The Ohio SSD cohort was approved by the University Hospital Institutional Review Board, affiliated with Case Western Reserve University. The mouse animal work (ArKO mice) was approved by the Howard Florey Institute Animal Ethics Committee, Australia (approval ID 08-070) and by the Stockholm’s South Animal Ethics committee, Sweden (approval ID S127-08). The rat hippocampal neuronal culture is under license number ESLH-2007-09085/Ym-23 to E. Castrén.

### Cohorts

See Table 1 for a more detailed description of each the six cohorts studied.

#### Finnish dyslexia cohort (FI, DYS)

Nineteen Finnish three-generation families (130 subjects; Table 1), of Caucasian origin and whose phenotypes were ascertained as previously described (Nopola-Hemmi et al. 2001), were genotyped (Kaminen et al. 2003; Anthoni et al. 2007).

The t(2;15)(p13;q22) family has been described phenotypically in detail previously (Nopola-Hemmi et al. 2000). Genomic DNA was obtained from blood lymphocytes using a standard non-enzymatic extraction method (Lahiri and Nurnberger 1991).

#### German dyslexia cohort (GER, DYS)

A total of 411 trios of German Caucasian origin (1,233 individuals totally; Table 1) were genotyped. All individuals, and in case of children younger than 14 years, their parents, gave written informed consent to participation in the study. The families were recruited from the Departments of Child and Adolescent Psychiatry and Psychotherapy at the Universities of Marburg and Würzburg. The diagnostic inclusion criteria and phenotypic measures have been described in detail previously (Schulte-Korne et al. 1996, 2001, 2007; Ziegler et al. 2005; Schumacher et al. 2006). Briefly, the diagnosis of dyslexia was based on the spelling score using the T distribution of the general population. Based on the correlation between IQ and spelling of 0.4 (Schulte-Korne et al. 2001), an anticipated spelling score was calculated. The child was classified as dyslexic if the discrepancy between the anticipated and the observed spelling score was at least one standard deviation. Probands and all siblings fulfilling the inclusion criteria were assessed with several psychometric tests. These tests targeted different aspects of the dyslexia, i.e. word reading, phonological awareness and short term memory (see Supplementary Table 9).

#### Colorado dyslexia cohort (CO, US, DYS)

This population was recruited through the Colorado Learning Disabilities Research Center and included 216 nuclear families with a total of 880 genotyped individuals (Table 1). Ascertainment and evaluation of this population has been described previously (DeFries et al. 1997). Briefly, families were selected through twins living in Colorado, at least one of whom had a history of reading problems by school report and confirmed by school records. Exclusion criteria included a full scale IQ score less than 80 and any sensory or medical problems that would interfere with reading. The twins and available siblings were given an extensive battery of assessments of reading, spelling, phonology, orthography, rapid naming, and intelligence. Measures used in the current analyses are given in Supplementary Table 9. DNA was obtained by extraction from blood, buccal swabs, or more recently from saliva samples. This sample set is made up of ~89% Caucasian, ~3% African-American, Asian, or Native American, and ~8% self-identified as mixed.

#### Georgia dyslexia cohort (GA, US, DYS)

Seventeen Caucasian families of US Caucasian origin and consisting of 57 subjects (Table 1) were studied. Families were recruited and referred through schools, physicians, and community announcements at the Center for Clinical and Developmental Neuropsychology (CCDN) at the University of Georgia. All qualifying families had at least one proband between the ages of 8 and 12 years with significant reading problems and no history of neurological impairment, traumatic brain injury, psychiatric disorders, or severe pre- and/or perinatal complications. All parents provided informed consent for the neuropsychological evaluation of themselves and their children. The test battery consisted of measures designed to assess intelligence, academic achievement, receptive and expressive language, phonological processing, memory, reading, spelling, visual-spatial ability, executive functioning, handedness, and social-emotional functioning (see Supplementary Table 9 for a full description of the tests used). Genomic DNA was obtained from buccal swabs by an NaOH extraction method (Walker et al. 1999). Whole genome amplification of the extracted DNA was performed by the improved primer preamplification method (I-PEP-L) (Hannelius et al. 2005).

#### Specific language impairment cohort (IA, US, SLI)

The Iowa cohort consisted of 573 participants of Caucasian origin, all members of an ongoing longitudinal study of children with SLI (Table 1) and a control group of typically developing age mates. The longitudinal cohort was obtained from a large population sample (*N* = 7,206) of monolingual English speaking kindergarten-age children from Iowa, who participated in a cross-sectional epidemiologic study of SLI. All children had normal hearing and no diagnosis of neurodevelopmental disorders. A description of the sampling methods for the original cross-sectional sample and selection of the longitudinal sample have been described previously (Tomblin et al. 1997, 2000). The members of this longitudinal cohort initially consisted of 604 children and slightly more than one-third presented with language impairment as 6-year-olds, and the remaining represented a random sample of typically developing age-mates. At the beginning of second grade, blood, saliva or buccal samples were obtained from the children and their parents. The phenotypic data for the current study were collected when the participants were in kindergarten and later in second grade. The speech sound production data were obtained when the children were in kindergarten. The remaining behavioral phenotypic measures including receptive and expressive language were obtained in second grade at which time the children had been receiving reading instruction for approximately 2 years (see Supplementary Table 9 for a full description of the measures used).

#### Speech sound disorder cohort (OH, US, SSD)

One-hundred-and-eighteen Caucasian families of US origin consisting of 550 subjects (80 affected with both SSD and dyslexia, 147 affected with only SSD, 41 affected with only dyslexia, 274 unaffected with either SSD or dyslexia and eight of unknown phenotype) were genotyped (Table 1). Probands were enrolled in speech-language therapy for a moderate to severe speech sound production disorder. Children were also required to have normal hearing, intelligence, and speech mechanism (adequate oral structures for producing speech sounds). An extensive battery of standardized speech sound production, receptive and expressive language, reading decoding and comprehension, spelling, oral-motor skills, memory, and phonological processing measures were administered to all probands and their siblings of 4 years of age and older (see Supplementary Table 9 for a listing of the specific measures). Genomic DNA was obtained from self-collected buccal swabs or blood draws.

#### Comparison of the SSD and SLI phenotypes to the dyslexia phenotypes

As seen in Supplementary Table 9, comparable domains were assessed for the Georgia dyslexia (GA, US, DYS), SSD (OH, US, SSD) and SLI (IA, US, SLI) cohorts where language, reading, spelling and phonological processing were measured.

### FISH and Southern blotting

For the mapping of the translocation breakpoints, 10 BAC clones from chromosome 2 (RP11-502A5, -419E14, -332A19, -89C12, -236I9, -521O14, -351F21, -1290B4, -548D17 and -513019; BACPAC Resource Center (BPRC) at Children’s Hospital Oakland Research Institute, Oakland, CA, USA) and 12 clones from chromosome 15 (RP11-10D13, -13H19, -56B16, -96N2, -108K3, -145A4, -209K10, -394B5, -430B1, -519C12, -522G20 and -540E17; Genome Systems, St Louis, MO, USA) were used as probes in FISH. Bacterial cultures and DNA isolation were performed according to standard protocols and probes were labeled by nick translation with FITC-dUTP (NEN Life Science Products, Boston, MA, USA), SpectrumOrange-dUTP (Vysis Inc, Downers Grove, IL), or biotin-14-dATP (detection with avidin conjugated FITC). FISH-analyses were performed according to standard protocols and the slides were analyzed on a Zeiss Axioplan 2 epifluorescence microscope (Carl Zeiss, Göttingen, Germany). Images were captured using a cooled CCD camera (Sensys Photometrics, München, Germany) and SmartCapture 2 (DigitalScientific Ltd., Cambridge, UK) or ISIS software (Metasystems GmbH, Altlussheim, Germany).

Genomic DNA (15 μg) from the individual carrying the translocation and from an unrelated control were digested with *Bam*HI, *Eco*RI, *Hin*dIII, *Kpn*I, *Sac*I, *Sca*I and *Sph*I and subjected to electrophoresis and Southern hybridization as previously described (Taipale et al. 2003). PCR-amplified genomic fragments from non-repetitive regions of the BAC clone RP11-108K3 were used as hybridization probes. PCR and labelling reactions were performed as previously described (Hannula-Jouppi et al. 2005).

Putative genes/exons from the 200 kb BAC clone spanning the breakpoint on chromosome 2 were in silico predicted using Genscan (genes.mit.edu/GENSCAN.html) and GrailEXP (grail.lsd.ornl.gov/grailexp). The expression of each of the 19 predicted genes/exons were tested by PCR on human cDNA libraries from fetal brain (cat. No. HL5504u, Clontech and cat. No. 052001b, Stratagene) and from leukocytes (cat. No. HL5509u and HL5019t, Clontech).

### Genotyping

In the Finnish (FI, DYS) and Georgia (GA, US, DYS) dyslexia cohorts, 20 SNPs (Fig. 2c, lowest part of the panel) were genotyped using matrix-assisted laser desorption/ionization time-of-flight (MALDI-TOF) mass spectrometry as previously described (Peyrard-Janvid et al. 2004). PCR assays and extension primers were designed using the SpectroDESIGNER software (Sequenom). The same procedure was applied to the German (GER, DYS) cohort for 16 of those 20 SNPs (all except rs934634, rs700519, rs749292 and rs3575192).

For the Colorado dyslexia (CO, US, DYS), the SSD (OH, US, SSD) and the SLI (IA, US, SLI) cohorts, genotype data for 16 of those 20 SNPs (all except rs700519, rs6493494, rs749292 and rs3575192) were successfully generated using the 5′ exonuclease TaqMan Assay by Design or Assays in Demand from Applied Biosystems (Foster City, CA, USA). Real-time PCR was conducted using the ABI 7700HT system. Genotypes were assigned with the SDS 2.0 software (Applied Biosystems).

CEPH genomic DNA, negative controls and replicates of some samples were included on each plate to assure consistency of the genotype calls. Discrepancies in genotype calls and Mendelian errors were identified using the PEDCHECK (O’Connell and Weeks 1998) and the MARKERINFO from the S.A.G.E. program package. All genotypes were independently confirmed by two investigators. Genotyping results were also cross-validated by duplicate genotyping of 10–96 samples across the different laboratories. Allele frequencies were also checked to match across the different data sets.

### Statistical methods

Testing for Hardy–Weinberg equilibrium was done via a Chi-squared goodness-of-fit test using only the founders to eliminate the non-independence owing to family data. Intermarker LD was visualized and pairwise *R* ^2^ values were determined using the Haploview v3.2 software (Barrett et al. 2005).

PDTPHASE v2.4 from the software package UNPHASED (Dudbridge 2003) was used to test for both single SNP and haplotype association with binary traits in all three populations, i.e. Finnish, German and North-American. This program is an implementation of the original PDT (Martin et al. 2000) but allowing missing data. Haplotypes were looked at in two- to four-marker sliding window.

A variance-component model developed for family-based association was used to assess single SNP significance of QTs in the GA, US, DYS and OH, US, SSD cohorts, as well as in both cohorts combined. This method assesses association between a marker and phenotype, while simultaneously estimating residual and multifactorial (polygenic, familial, and marital) variance components. Age was found to be significant in both populations and therefore was included in the baseline model as a covariate. At each SNP and for each trait, we tested for an additive, a dominant or a recessive allele effect. These three tests are correlated with each other and, because any two of these null hypotheses imply the third, they effectively count as two independent tests (Elston et al. 1999). Therefore, in each population and for each trait, the total number of independent tests performed is equal to twice the number of SNPs genotyped. To account for these multiple tests when determining allelic association to a trait, Sidak’s correction was used (Sidak 1967).

Because the same reading test (WRMT-R, see Supplementary Table 9) was administered to participants in the GA, US, DYS and the OH, US, SSD cohorts, and the definition of dyslexia used to classify participants as affected was identical across the two cohorts, we combined *p*-values from tests of allelic association using Fisher’s method (Fisher 1948).

### Evolutionary analysis of the *CYP19A1* genomic sequence

Evolutionary comparison of the ~123 kb *CYP19A1* genomic region, covering the full promoter as well as the coding region of the gene was performed using the GenomeVISTA browser (pipeline.lbl.gov/cgi-bin/GenomeVista). The human sequence (49,285,000–49,420,000 bp on chromosome 15, NCBI Build 36.1) was aligned with the genomic sequences of dog (*Canis familiaris*), mouse (*Mus musculus*), opossum (*Monodelphis domestica*) and frog (*Xenopus tropicalis*).

### Evolutionary analysis of the *CYP19A1* coding sequence

Chimpanzee (*Pan troglodytes*), pigmy chimpanzee (*Pan* *paniscus*), gorilla (*Gorilla gorilla*) and orangutan (*Pongo* *pygmaeus*) (DNA samples kindly provided by Kathrin Koehler, Max Planck Institute of Evolutionary Anthropology, Leipzig, Germany) orthologues were screened for variations by direct sequencing with human-specific, intronic primers (all primer sequences available on request). All nine coding exons and the brain-specific exon/promoter I.f of *CYP19A1*, including 100 bp of flanking sequence, were PCR-amplified in 25 μl reactions containing 20 ng DNA, 1.5 mM MgCl_2_, 0.4 mM of each dNTP, 1 μM of each primer and 0.03 U/μl of HotStarTaq DNA polymerase (Qiagen). We used a touch-down protocol with 42 cycles of amplification with 1°C decrease in annealing temperature at each round. The amplification started with two cycles at 63 and 62°C respectively, followed by three cycles at each temperature between 61 and 56°C, and ending by 10 cycles at 55 and 54°C, respectively. PCR cycles had an initial denaturation at 95°C for 15 min; 30 s for each annealing and 30 s to 1 min 30 s elongation at 72°C; and a final extension of 10 min at 72°C. PCR products were dephosphorylated by 0.4 U/μl shrimp alkaline phosphatase (Amersham Biosciences/GE) and 2 U/μl exonuclease I (New England BioLabs), and were further sequenced using DYEnamic ET Dye terminator kit (Amersham Biosciences/GE) following the manufacturer’s instructions. Each fragment was sequenced from both directions using the same primers as in the PCR reaction. Purified sequencing products were resolved using a MegaBACE 1000 instrument and MegaBACE long-read matrix (Amersham Biosciences/GE), visualized using the Sequence Analyzer v3.0 software (Amersham Biosciences/GE), and assembled and analyzed using the Pregap and Gap4 software (www.cbi.pku.edu.cn/tools/staden), comparing to the sequence NT_010194, build 36 (www.ncbi.nih.gov). Sequences were verified visually by two independent readers.

Evolutionary analysis of the *CYP19A1* coding sequence was performed with a likelihood ratio test using the CODEML program of the paml3.15 package (Yang 1997); the dog (*Canis familiaris*) XP_544678 sequence was used as the outgroup in the analysis.

### EMSA

Fragments of 30 bp in length and for each allele of the three SNPs studied were designed as probes (the sequences can be obtained on request). EMSA was performed according to standard protocols. The binding reactions were performed by pre-incubating 10 μg nuclear- or total cell extracts from the neuroblastoma cell line SH-SY5Y, with 0.5 μg of poly (dI-dC), 10 mM DTT and 100 mM NaCl. ^32^P-end-labeled double-stranded probes were added, and the mixture was incubated for 20 min at room temperature (RT). For the supershift assays, 4 μg of TFII-I or Elk-1 antibody (cat. Nos. sc-9943 X and sc-355 X; Santa Cruz Biotechnology, Inc.) were added to the reaction and incubated for another 20 min at RT. For the competition assays, a 100- and 200-mol excess of non-labeled oligonucleotide was added and incubated for 30 min at RT prior to addition of the labeled probe. The samples were electrophoresed on 5% non-denaturing polyacrylamide gels in 1× TBE (0.09 M Tris–borate, 2 mM EDTA) at 150 V. The radioactive pattern was visualized by autoradiography and quantified by PhosphorImager scanning (Fuji Photo Film Co., Ltd., Stamford, CT, USA). Transcription factor binding sites for both alleles of each SNP were predicted by TESS (www.cbil.upenn.edu/cgi-bin/tess/tess).

### Expression analysis

Ready-made TaqMan gene expression assays for *CYP19A1* (Hs00240671_m1), *DYX1C1* (Hs00370049_m1), *DCDC2* (Hs00393203_m1), *KIAA0319* (Hs00207788_m1), *ROBO1* (Hs00268049_m1), *MRPL19* (Hs00608519_m1), *C2ORF3* (Hs00162632_m1), and *18S rRNA* (4319413E) were purchased from Applied Biosystems. We assayed expression levels for these genes in total RNA from nine different regions of adult human brain: thalamus, hypothalamus, frontal-, occipital-, parietal-, temporal cortex (cat. Nos. 6762, 6864, 6810, 6812, 6814, 6816, Ambion); hippocampus, paracentral-, postcentral gyrus (cat. Nos. 636565, 636574, 636573, Clontech); as well as from whole adult brain (cat. No. 636530, Clontech). For each tissue, three independent cDNA syntheses (500 ng total RNA per reaction) were performed using the SuperScript III first-strand synthesis kit (cat. No. 18080-051, Invitrogen). From each cDNA synthesis, quantitative real-time PCR was performed in quadruplets, using 5–50 ng of RNA per gene assay and run on ABI PRISM 7700 Sequence Detection PCR System (Applied Biosystems). All assays were performed in 10 μl reactions according to the manufacturer’s instructions. Relative standard expression curves were drawn for 18S rRNA and all tested genes. Relative quantification of the data was performed using the comparative threshold cycle (Ct) method (Sequence Detection System bulletin 2, Applied Biosystems) adjusting the Ct values to 18S rRNA.

### Generation of ArKO mice brain sections

ArKO mice were generated through breeding heterozygous mice (Fisher et al. 1998). ArKO +/− female mice were mated overnight with ArKO +/− males and inspected at 9:00 a.m. on the following day for the presence of vaginal plug. Noon of this day was assumed to correspond to E0.5. All animals were housed in the animal-care facility with a 12 h light/12 h dark photo-period and given free access to tap water and rodent chow. To obtain E17.5 embryos, pregnant mice were anaesthetized deeply with CO_2_ and perfused with PBS followed by 4% paraformaldehyde (in 0.1 M PBS, pH 7.4). Embryos were taken out and put on ice, and brains were dissected and postfixed in the same fixative overnight at 4°C. For the 5-month-old mice, mice were perfused individually with PBS followed by 4% paraformaldehyde, and brains were then removed and postfixed overnight. Sex was determined after direct visual inspection of the gonads with a dissecting microscope, and the tail and limbs were removed from each embryo for genotyping. After fixation, brains were processed for either paraffin (6 μm) or frozen (30 μm) sections.

### Immunohistochemistry

The paraffin-embedded embryonic ArKO mice brain sections were dewaxed in xylene and rehydrated through graded alcohol to H_2_O. Nissl staining (0.25% thionin) was used to examine the histology of embryonic brains with light microscopy. For the immunohistochemistry study, paraffin sections were processed for antigen retrieval with 10 mM citrate buffer (pH 6.0), and then processed in the same manner as the frozen sections. Briefly, sections were blocked for 30 min with 1% H_2_O_2_ followed by 10% normal serum, rinsed three times with PBS, and incubated overnight at 4°C with the following antibodies: polyclonal rabbit anti-EGF (Santa Cruz Biotechnology) and mouse anti-NeuN (MAB377, Chemicon) were used at 1:100, and the anti-parvalbumin mouse monoclonal antibody (Swant, Switzerland) was used at 1:1,000. After washing, sections were incubated with Cy3-conjugated anti-rabbit or Cy3-conjugated anti-mouse antibodies (Jackson ImmunoResearch) in 1:200 dilutions for 2 h at RT. Parvalbumin-positive cells were counted on images in an area of 200 × 200 μm in the somatosensory cortex in coronal sections (three animals per condition, 10 images each). All pictures were location-matched between WT and the ArKO mice. Statistical analysis was performed using Student’s *t* test.

### Neuron quantification

Mice were anaesthetized (Pentobarbitol; 0.2 ml i.p./20 g) and perfused through the heart via the ascending aorta with 20 ml Ca^2+^-free Tyrode’s buffer (37°C), followed by 20 ml of a mixture of 4% paraformaldehyde and 0.2% picric acid diluted in 0.16 M phosphate buffer (pH 6.9) and 50 ml of the same fixative at 4°C for 5 min. Brains were removed and post-fixed in the same fixative for 90 min at 4°C, and finally immersed for 48 h in 10% sucrose dissolved in phosphate buffered saline (PBS, pH 7.4) containing 0.01% sodium azide (Sigma, St. Louis, MO, USA) and 0.02% Bacitracin (Sigma) at 4°C, before rapid freezing by dry ice and sectioned at 20 μm (cryostat; Leica CM 1850). The neuronal density of the number of NeuN immunoreactive cell bodies in the somatosensory cortex was estimated by systemic random sampling of four sections per animal (ArKO, *n* = 4; WT, *n* = 5). Images of the somatosensory cortices were scanned using an Olympus BX-51 fluorescent microscope at ×100 magnification using the TRITC UV filter. A counting grid was placed over the somatosensory cortex with dimensions of each counting frame being 251.5 × 83.2 μm = 0.02 mm^2^. Cells falling within each frame were quantified. For counting purposes, NeuN immunoreactive cell bodies falling on the upper and right boundaries of the counting frames were counted while NeuN immunoreactive cell bodies that fell on the lower and left boundaries of each frame were ignored.

### Commissural measurements

Five two-month-old male ArKO mice and five WT littermates were used to measure the commissures. The commissures were visualized by cutting the fixed brain in half at the mid-sagittal plane, and staining the myelinated structures with gold chloride (Wahlsten et al. 2003). Digital images were obtained with Olympus SZx9 Research Stereo microscope and Olympus DP70 digital microscope camera, and the areas of CC, HC and anterior commissure (AC) were measured with the ImageJ program (Abràmoff et al. 2004).

### Process outgrowth of rat hippocampal neurons

Hippocampal neuronal cultures were prepared from the brains of E17 rat fetuses (Brewer and Cotman 1989). Briefly, the hippocampi were dissected, the meninges removed and the neurons dissociated in single-cell suspension with papain (0.5 mg/ml) digestion and mechanical trituration. The cells were centrifuged, suspended in DMEM containing Glutamax I and supplemented with 10% heat inactivated fetal bovine serum (FBS), 100 U/ml penicillin, and 100 μg/ml streptomycin (DMEM medium; Gibco BRL) and 100,000 cells/well on 12-well plates were plated onto glass coverslips coated with 0.5 mg/ml poly-l-ornithine (Sigma) and 10 mg/ml laminin (Invitrogen). The cells were cultured in neurobasal medium (Gibco), without phenol red supplemented with B27 (Gibco), penicillin–streptomycin (Euroclone) and l-glutamine (Euroclone) at 37°C in 5% CO_2_. After 24 h in vitro, cells were treated with only solvent; or testosterone (20 nM, Fluka) or testosterone (20 nM) and letrozole (100 nM, Advanced Technology and Industrial Co., Hong Kong); or testosterone (20 nM), letrozole (100 nM) and 17β-estradiol (1 nM, Sigma); or 17β-estradiol (1 nM) only. After 3–4 days of in vitro culture, cells were fixed with 4% paraformaldehyde and immunostained with the neuronal marker TuJ1 (Covance). Confocal microscope pictures were taken (Zeiss Axioplan 2, Pascal software) and total neurite outgrowth per neuron was measured using Image-Pro Plus software tracing all processes (*N* ~ 70/group).

## Electronic supplementary material

Below is the link to the electronic supplementary material.

Supplementary material 1 (DOCX 63 kb)
