# Supplementary material for: The Aromatase Gene CYP19A1: Several Genetic and Functional Lines of Evidence Supporting a Role in Reading, Speech and Language
Source: Behav Genet. 2012 Mar 17;42(4):509–27. doi: 10.1007/s10519-012-9532-3 (PMC3375077; doi:10.1007/s10519-012-9532-3)
Supplement: Supplementary file 1 — Supplementary material 1 (DOCX 63 kb) [file 10519_2012_9532_MOESM1_ESM.docx]

**SUPPLEMENTARY INFORMATION**

**SUPPLEMENTARY TABLES**

**Suppl. Table 1**. **Sequence analysis of *CYP19A1* exons in chimpanzee, pigmy chimpanzee, gorilla and orangutan**

| **Exon** | **DNA change**  **Human/Primate^** | **Amino acid change** | | | |
| --- | --- | --- | --- | --- | --- |
|  |  | **Chimpanzee** | **Pigmy chimpanzee** | **Gorilla** | **Orangutan** |
| *I.f* | *T/C* | *C/C* | *C/C* | *C/C* | *C/C* |
| *I.f* | *C/T* |  |  | *C/T** |  |
| *I.f* | *A/C* |  | *C/C* |  |  |
| 2 | 24G/A |  |  |  | Pro8Pro |
| 2 | 27A/G |  |  |  | Ile9Met |
| 2 | 82G/A |  |  |  | Val28Ile |
| 3 | 186C/T |  |  |  | His62His |
| 3 | 210C/T | Ile70Ile | Ile70Ile |  |  |
| 4 | 297G/A |  | Lys99Lys* |  |  |
| 4 | 417G/T |  |  | Glu139Asp* | Glu139Asp |
| 5 | 459A/G |  |  | Ser153Ser | Ser153Ser |
| 5 | 465C/T |  |  |  | Pro155Pro |
| 5 | 477T/C |  |  |  | Arg159Arg |
| 5 | 546G/A |  |  |  | Ser182Ser |
| 5 | 552T/C |  |  |  | Tyr184Tyr |
| 5 | 602C/T |  |  |  | Thr201Met |
| 7 | 765A/G | Ile255Met* |  |  |  |
| 7 | 855A/G |  |  |  | Ala285Ala* |
| 9 | 1101G/A |  |  |  | Gln367Gln |
| 9 | 1107C/T |  |  |  | Val369Val |
| 9 | 1143T/C |  |  | Asp381Asp |  |
| 9 | 1149C/T |  |  | Ile383Ile |  |
| 9 | 1176A/G |  |  |  | Thr392Thr |
| 9 | 1221C/T |  |  |  | Phe407Phe |
| 10 | 1287A/G |  | Pro429Pro |  |  |
| 10 | 1476T/C |  | Phe492Phe |  |  |

^Nucleotide variations between human and primate *CYP19A1* are listed according to the transcription start site, *heterozygous changes.

# Suppl. Table 2. Divergence from the human *CYP19A1* sequence

| **Primate** | **Nucleotide** | | **Amino acid** | | | |
| --- | --- | --- | --- | --- | --- | --- |
|  |  | **%** | **nonsyn** | **%** | **syn** | **%** |
| Chimpanzee | 2/1509 | 0.13 | 0/503 | 0 | 1/503 | 0.20 |
| Pigmy chimpanzee | 4/1509 | 0.27 | 0/503 | 0 | 3/503 | 0.60 |
| Gorilla | 4/1509 | 0.27 | 0/503 | 0 | 3/503 | 0.60 |
| Orangutan | 19/1509 | 1.26 | 4/503 | 0.80 | 11/503 | 2.19 |

| **Model** | ***p*** | ***l*** | **κ** | **ωH** | **ωC** | **ωP** | **ωG** | **ωO** | **ωD** |
| --- | --- | --- | --- | --- | --- | --- | --- | --- | --- |
| A. One ratio: ωH = ωC = ωP = ωG = ωO = ωD | 12 | -2796.19 | 4.238 | 0.1835 | 0.1835 | 0.1835 | 0.1835 | 0.1835 | 0.1835 |
| B. Two ratios: ωH = ωC = ωP = ωG = ωO, ωD | 13 | -2795.59 | 4.224 | 0.1106 | 0.1106 | 0.1106 | 0.1106 | 0.1106 | 0.1106 |
| C. Three ratios: ωH = ωC = ωP, ωG = ωO, ωD | 14 | -2792.72 | 4.162 | 0.0001 | 0.0001 | 0.0001 | 0.0266 | 0.0266 | 0.2047 |
| D. Three ratios: ωH, ωC = ωP = ωG = ωO, ωD | 14 | -2793.32 | 4.148 | 1.0766 | 0.0162 | 0.0162 | 0.0162 | 0.0162 | 0.2032 |
| E. Free ratios | 21 | -2790.43 | 4.089 | 0.0001 | 0.0001 | 0.0001 | 0.0001 | 0.0001 | 0.0647 |

# Suppl. Table 3. Log likelihood values and parameter estimates under different models

*p,* number of parameters; *l,* log likelihood value; κ, amino acid transition/transversion ratio; ωH, *dN*/*dS* for the human branch; ωC, *dN*/*dS* for the chimpanzee branch; ωP, *dN*/*dS* for the pigmy chimpanzee branch; ωG, *dN*/*dS* for the gorilla branch; ωO, *dN*/*dS* for the orangutan branch; ωD, *dN*/*dS* for the dog branch. Tree used in analysis: ((((human, (chimpanzee, pigmy chimpanzee)), gorilla), orangutan), dog).

###### Suppl. Table 4. Likelihood ratio tests for hypotheses

| **Models** | **χ^2^** | ***df*** | ***P*** |
| --- | --- | --- | --- |
| A vs. B | 1.205 | 1 | 0.272 |
| A vs. C | 6.938 | 2 | 0.031 |
| A vs. D | 5.744 | 2 | 0.057 |
| B vs. C | 5.733 | 1 | 0.017 |
| B vs. D | 4.539 | 1 | 0.033 |

Suppl. Table 5. Mid-sagittal areas of corpus callosum, the anterior and hippocampal commissures in aromatase knock-out (ArKO) and wild-type (WT) mice.

|  |  | **AREA (mm^2^)**  **Mean ± SD** | | | |
| --- | --- | --- | --- | --- | --- |
|  | N | **CC** | **HC** | **AC** | **Total** |
| WT | **5** | 0.836 ± 0.085 | 0.208 ± 0.056 | 0.100 ± 0.004 | 1.145 ± 0.138 |
| ArKO | **5** | 0.855 ± 0.077 | 0.194 ± 0.014 | 0.103 ± 0.010 | 1.151 ± 0.087 |

## CC, corpus callosum; HC, hippocampal commissure; AC, anterior commissure.

### Suppl. Table 6. Quantitative trait (QT) analysis of the Ohio speech sound disorder cohort (OH, US, SSD)

| OH, US, SSDallelic association | | **SNPs in *CYP19A1*** | | | | | | | | | | | | | | | |
| --- | --- | --- | --- | --- | --- | --- | --- | --- | --- | --- | --- | --- | --- | --- | --- | --- | --- |
|  |  |  |  |  |  |  |  |  |  |  |  |  |  |  |  |  |  |
| Phenotype | Measure* | **rs934634**  **C/T** | **rs10046**  **C/T** | **rs2289105**  **C/T** | **rs8034835 A/G** | **rs2899472 A/G** | **rs1065778 A/G** | **rs700518**  **A/G** | **rs767199**  **A/G** | **rs11632903 C/T** | **rs1902586 A/G** | **rs936306**  **C/T** | **rs2470176 A/G** | **rs730154**  **C/T** | **rs2470152 A/G** | **rs1004984**  **C/T** | **rs2470144 A/G** |
| Reading | Reading Comprehension |  |  |  |  |  |  |  |  | 0.005 |  |  |  |  |  |  |  |
| Phonological processing | Repetition of multi-syllabic words |  | 0.005 | **0.002** | 0.007 |  | 0.006 | 0.009 | 0.006 |  |  | 0.02 | 0.03 | **0.001** | **0.002** |  |  |
|  | Repetition of nonsense words | **0.001** | **0.00005** | 0.005 | **0.00004** | 0.009 |  | 0.02 | 0.006 | **0.001** |  | 0.03 | 0.04 | 0.05 | 0.003 |  |  |
| Language | Listening Comprehension |  |  |  |  | 0.02 |  |  |  |  |  |  |  |  | 0.02 |  |  |
|  | Vocabulary |  | **0.000007** | **0.00003** |  |  |  |  |  |  |  | 0.003 | 0.02 |  |  |  |  |
| Memory | Digit Span |  | 0.04 | 0.02 | 0.004 |  | 0.02 |  | 0.03 |  |  |  |  |  |  |  |  |
|  | Sentence Imitation | 0.03 |  |  |  | 0.02 |  |  |  |  |  |  |  |  |  |  |  |
| Oral motor skills | Rate of repetition of single syllables |  |  | 0.04 |  |  | 0.05 | 0.03 |  |  |  |  |  |  |  | **0.000004** |  |
|  | Rate of repetition of double syllables | 0.02 | 0.05 | **0.000002** | 0.007 | 0.002 | **0.00004** | **0.0000001** |  | 0.004 | 0.002 |  |  |  |  | 0.007 |  |
| Non-verbal cognition | Performance IQ | 0.007 |  |  | **0.001** |  |  |  |  |  |  | 0.04 |  |  |  |  |  |

*The psychometric test battery used is described in Suppl. Table 9.

### Suppl. Table 7. Quantitative trait (QT) analysis of the Georgia dyslexia cohort (GA, US, DYS)

| GA, US, DYSallelic association | | **SNPs in *CYP19A1*** | | | | | | | | | | | | | | | | | | |
| --- | --- | --- | --- | --- | --- | --- | --- | --- | --- | --- | --- | --- | --- | --- | --- | --- | --- | --- | --- | --- |
| Phenotype | Measure* | **rs934634**  **C/T** | **rs10046**  **C/T** | **rs2289105**  **C/T** | **rs700519**  **C/T** | **rs8034835 A/G** | **rs2899472 A/G** | **rs1065778 A/G** | **rs700518**  **A/G** | **rs767199**  **A/G** | **rs6493494 A/G** | **rs749292**  **A/G** | **rs11632903 C/T** | **rs1902586 A/G** | **rs936306**  **C/T** | **rs2470176 A/G** | **rs2470152 A/G** | **rs3575192 A/G** | **rs1004984**  **C/T** | **rs2470144 A/G** |
| Reading | GORT-3 Passages | 0.01 | 0.002 | 0.01 |  | 0.002 |  | 0.003 | **0.0004** |  |  |  |  |  |  |  | 0.04 | 0.05 |  |  |
|  | GORT-3 Passage Comprehension |  | 0.05 |  |  | 0.007 |  | **0.0006** |  |  |  |  |  |  |  |  | 0.01 |  |  |  |
| Phonological  processing | C-TOPP Elision | 0.008 |  |  |  |  |  |  |  |  |  |  |  |  |  |  | 0.05 |  | 0.01 | 0.01 |
| Language | Boston Naming Test |  |  | 0.01 |  |  | **0.001** | 0.002 | 0.02 |  |  |  |  |  |  |  | 0.003 |  |  |  |
| Spelling | WRAT-Spelling | 0.01 | 0.002 | 0.05 |  | 0.02 |  | 0.04 |  |  |  | 0.02 | 0.01 |  |  |  |  |  | 0.03 |  |
| Phoneme awareness | Phoneme Reversal Task | 0.03 |  |  |  |  |  | 0.02 |  |  |  | 0.03 | 0.03 |  |  |  |  |  | 0.01 | 0.04 |
| Cognition | Full Scale IQ | 0.04 | 0.02 | 0.02 |  | 0.02 |  | 0.02 | 0.04 |  |  |  |  |  |  |  |  |  |  |  |

### *The psychometric test battery used is described in Suppl. Table 9. Three models were tested for each SNP covariate: an additive model (0=1/1, 1=1/2, 2=2/2), a recessive model (1=1/1, 0=1/2, 0=2/2), and a dominant model (0=1/1, 1=1/2, 1=2/2). All models were adjusted for age. Alleles 1 and 2 are designated at the top of the column as 1/2. For each SNP and trait the three models were compared to the baseline model (without the SNP covariate) using a Likelihood Ratio Test (LRT). The most significant p-values (for all p<0.10) of the LRT statistics for each SNP and trait are presented. P-values that remain significant at the 0.05 level after correction for multiple testing are in bold. Complete information on each model is available from the authors.

### Suppl. Table 8. Pooled analysis of quantitative phenotypes in the Georgia dyslexia (GA, US, DYS) and the Ohio speech sound disorder (OH, US, SSD) cohorts

| Cohort | **Phenotype** | **Measure*** | **rs934634**  **C/T** | **rs10046**  **C/T** | **rs2289105**  **C/T** | **rs700519**  **C/T** | **rs8034835 A/G** | **rs2899472 A/G** | **rs1065778 A/G** | **rs700518**  **A/G** | **rs767199**  **A/G** | **rs6493494 A/G** | **rs749292**  **A/G** | **rs11632903 C/T** | **rs1902586 A/G** | **rs936306**  **C/T** | **rs2470176 A/G** | **rs730154**  **C/T** | **rs2470152 A/G** | **rs3575192 A/G** | **rs1004984**  **C/T** | **rs2470144 A/G** |
| --- | --- | --- | --- | --- | --- | --- | --- | --- | --- | --- | --- | --- | --- | --- | --- | --- | --- | --- | --- | --- | --- | --- |
| GA, US, DYS | Timed Naming | C-TOPP Rapid Naming |  | 0.03 | 0.03 |  |  |  | 0.02 |  |  |  |  |  |  | 0.006 |  |  |  | 0.05 |  |  |
| OH, US, SSD |  |  |  |  | 0.05 |  |  |  |  |  |  |  |  | 0.02 | 0.003 |  |  | **0.0003** |  |  |  |  |
| Combined^ |  |  |  |  | 0.01 |  |  |  |  |  |  |  |  |  |  |  |  |  |  |  |  |  |
| GA, US, DYS | Reading | WRMT-R Word Attack | **0.00009** | **0.0007** |  |  | 0.002 |  | 0.003 | 0.006 | 0.05 |  | 0.03 |  |  |  |  |  | 0.02 |  |  | 0.02 |
| OH, US, SSD |  |  |  |  | 0.006 |  |  |  | 0.03 | 0.03 | 0.04 |  |  |  |  |  |  |  |  |  |  |  |
| Combined^ |  |  |  |  |  |  |  |  | 0.0009 | 0.002 | 0.01 |  |  |  |  |  |  |  |  |  |  |  |
| GA, US, DYS |  | WRMT-R Word Identification | 0.02 | 0.002 | 0.006 |  | **0.0003** |  | 0.01 | 0.008 |  |  |  | 0.04 |  |  |  |  | 0.02 |  |  | 0.003 |
| OH, US, SSD |  |  |  | 0.02 | 0.01 |  |  |  |  |  | 0.01 |  |  |  |  |  |  |  |  |  |  |  |
| Combined^ |  |  |  | 0.0005 | 0.0006 |  |  |  |  |  |  |  |  |  |  |  |  |  |  |  |  |  |

### *The psychometric test battery used is described in Suppl. Table 9; ^P-values determined using Fisher’s pooling method. Three models were tested for each SNP covariate: an additive model (0=1/1, 1=1/2, 2=2/2), a recessive model (1=1/1, 0=1/2, 0=2/2), and a dominant model (0=1/1, 1=1/2, 1=2/2). All models were adjusted for age. Alleles 1 and 2 are designated at the top of the column as 1/2. For each SNP and trait the three models were compared to the baseline model (without the SNP covariate) using a Likelihood Ratio Test (LRT). The most significant p-values (for all p<0.10) of the LRT statistics for each SNP and trait are presented. P-values that remain significant at the 0.05 level after correction for multiple testing are in bold. Complete information on each model is available from the authors.

## Suppl. Table 9. Measures administered to participants in the German (GER, DYS), the Colorado (CO, US, DYS) and the Georgia (GA, US, DYS) dyslexia cohorts, the Ohio speech sound disorder (OH, US, SSD) cohort and the Iowa specific language impairment (IA, US, SLI) cohort

| Domain | German dyslexia (GER, DYS) | Colorado dyslexia  (CO, US, DYS) | Georgia dyslexia  (GA, US, DYS) | Ohio speech sound disorder  (OH, US, SSD) | Iowa specific language impairment  (IA, US, SLI) |
| --- | --- | --- | --- | --- | --- |
| Articulation (SSD) | None | None | None | Goldman-Fristoe Test of Articulation (GFTA) (23)*^  Khan-Lewis Phonological Analysis (KLPA‑2) (24)*  Conversational speech sample analysis (PCC) (25)*^  For parents and older children: Articulation test (26), Multisyllabic Word Repetition, Nonsense Word Repetition (NSW), Pig Latin Task (27) | Articulation subtest of Test of Language Development-Primary 2^nd^ Edition (TOLD-P: 2) (44)*^ |
| Oral-motor | None | None | None | Oral and Speech Motor Control Protocol (28)*  Fletcher Time-by-Count (29)^ | None |
| Phonological skills | Phoneme segmentation, Phoneme deletion, Phoneme reversal tests (1) | Phoneme transposition (8)  Phoneme deletion (9)  Nonword reading (8) | Comprehensive Test of Phonological Processing (CTOPP) (13) | CTOPP (13)  Segmentation Task (30)*  Multisyllabic Word Repetition (31)*^  NSW (30)*^  Rapid Automatized Naming- Colors (RAN‑C) (32)*^  Elision Task (Torgesson, personal communication)^ | Syllable/phoneme deletion task (45)  Non-word repetition task (46) |
| Language | None | None | Boston Naming Test (BNT) (14)  Clinical Evaluation of Language Fundamentals:3^rd^ Edition (CELF-3) (15)  Listening comprehension and memory for sentences; subtest of Johnson Test of Cognitive Ability-Revised (WJ-R) (16) | Clinical Evaluation of Language Fundamentals- Preschool (CELF-P) (33)*  TOLD-P: 3 (34)*^  CELF-3 (15)^  Peabody Picture Vocabulary Test- 3^rd^ Edition (PPVT-III) (35)*^  Expressive One Word Picture Vocabulary Test (EOWPVT) (36)*^ | CELF-P (33)*  Comprehensive Receptive and Expressive Vocabulary Test (CREVT) (47)  PPVT-R (48)*^ |
| Reading | Word reading test (accuracy and reading speed) (2) | Timed word reading (9)  PIAT word recognition (10)  PIAT reading comprehension (10) | Gray Oral Reading Test-3^rd^ Edition (GORT‑3) (17)  Woodcock Reading Mastery Tests-Revised (WRMT-R) (18) | WRMT-R (Word ID and Word Attack) (37)^  Wechsler Individual Achievement Test (WIAT- Reading Comprehension) (38)^ | GORT‑3 (17)  WRMT-R (18) |
| Spelling | German spelling test (writing to dictation) (3) | PIAT Spelling (10)  Orthographic coding (9) | Wide Range Achievement Test-3^rd^ Edition (WRAT-3) (14) | Test of Written Spelling- 3^rd^ Edition (TWS‑3) (39)^  Test of Written Language- 2nd Edition (TOWL‑2) (40)^ | None |
| Intelligence | Culture Fair Test (CFT-1 (4); or CFT-20 (5)) | WISC-IV (11)  WAIS-III (12) | Wechsler Abbreviated Scale of Intelligence (WASI) (19) | Wechsler Preschool and Primary Scale of Intelligence-Revised (WPPSI-R) (41)*  Wechsler Intelligence Scale for Children-3^rd^ Edition (WISC-III) (42)^ | WISC-III (42)^ |
| Visual-perceptual skills | None | None | Visual closure subtest of Woodcock (WJ-R) (16) | None | None |
| Handedness | None | None | Edinburgh Handedness Inventory (20) | None | None |
| Memory | Digit span test from the HAWIK-R (6) (German adaptation of the WISC-R (7) | None | Children’s Memory Scale (CMS) (21) | Digit span subtest of WISC-III (42)^  Test of Auditory-Perceptual Skills-Revised (43) | None |
| Visual-spatial processing | None | None | Arrows subtest of NEPSY (22) | None | None |

*preschool test battery; ^school age test battery

**SUPPLEMENTARY REFERENCES**

1. Schulte-Körne, G., Ziegler, A., Deimel, W., Schumacher, J., Plume, E., Bachmann, C., Kleensang, A., Propping, P., Nöthen, M.M., Warnke, A. *et al.* (2007) Interrelationship and familiality of dyslexia related quantitative measures. *Ann Hum Genet,* **71,** 160-175.

2. Landerl, K., Wimmer, H. and Moser, E. (1997*)* Salzburger Lese- und Rechtschreibtest [The Salzburg reading and spelling test]. Hans Huber, Bern.

3. Brähler, E., Holling, V., Leutner, D. and Petermann, F. (2002) Handbuch psychologischer und pädagogischer tests.

4. Weiss, R.H. and Osterland, J. (1997) Grundintelligenztest Skala 1 (CFT 1). Hogrefe, Göttingen.

5. Weiss, R.H. (1998) Grundintelligenztest Skala 2 (CFT 20). Hogrefe, Göttingen.

6. Tewes, U. (1983) *HAWIK-R.* Hamburg-Wechsler Intelligenztest für Kinder. Hans Huber, Bern.

7. Wechsler, D. (1974) Wechsler Intelligence Scale for Children - Revised (WISC-R). The Psychological Corporation, San Antonio, TX, USA.

8. Gayan, J., & Olson, R.K. (2001). Genetic and environmental influences on orthographic and phonological skills in children with reading disabilities. *Dev Neuropsych,* **20**, 487-511.

9. Olson, R., Forsberg, H., Wise, B., & Rack, J. (1994). Measurement of word recognition, orthographic, and phonological skills. In G.R. Lyon (Ed.) Frames of reference for the assessment of learning disabilities: New views on measurement issues (pp. 243-277). Baltimore: Paul H. Brookes Publishing Co.

10. Peabody Individual Achievement Test–Revised-Normative Update (PIAT-R/NU; Markwardt, 1989, 1998)

11. Wechsler, D. (2003). Examiners’ manual: Wechsler Intelligence Scale for Children-Fourth Edition. San Antonio, TX: The Psychological Corporation.

12. Wechsler, D. (1997). Wechsler Adult Intelligence Scale–3rd Edition. San Antonio, TX: The Psychological Corporation.

13. Wagner, R.K., Torgesen, J.K. and Rashotte, C.A. (1999) Comprehensive Test of Phonological Processing (C-TOPP). Pro-Ed, Austin, TX, USA.

14. Goodglass, H., Kaplan, E., Weintraub, S. and Ackerman, N. (1983) The Boston Naming Test. Lea & Febiger, Philadelphia, PA, USA.

15. Semel, E., Wiig, E.H. and Secord, W. (1995) Clinical Evaluation of Language Fundamentals - 3rd Edition (CELF-3). The Psychological Corporation, San Antonio, TX, USA.

16. Woodcock, R.W. and Johnson, M.B. (1989) Woodcock-Johnson Psycho-Educational Battery - Revised. DLM Teaching Resources, Allen, TX, USA.

17. Wiederholt, J.L. and Bryant, B.R. (1992) Gray Oral Reading Test - 3, Third edition. Pro-Ed., Austin, Texas, USA.

18. Woodcock, R.W. (1998) Woodcock Reading Mastery Tests - Revised: Normative Update. American Guidance Services, Circle Pines, MN, USA.

19. Wechsler, D. (1999) Wechsler Abbreviated Scale of Intelligence (WASI). The Psychological Corporation, San Antonio, TX, USA.

20. Oldfield, R.C. (1971) The assessment and analysis of handedness: the Edinburgh inventory. *Neuropsychologia,* **9,** 97-113.

21. Cohen, M.J. (1998) Children's Memory Scale (CMS). The Psychological Corporation, San Antonio, TX.

22. Korkman, M., Kirk, U. and Kemp, S. (1997). The Psychological Corporation, San Antonio, Texas, USA.

23. Goldman, R. and Fristoe, M. (1986) The Goldman-Fristoe Test of Articulation (GFTA). American Guidance Services, Circle Pines, MN, USA.

24. Khan, L. and Lewis, N. (1986) Khan-Lewis Phonological Analysis (KLPA). American Guidance Services, Circle Pines, MN, USA.

25. Shriberg, L.D., Austin, D., Lewis, B.A., McSweeny, J.L. and Wilson, D.L. (1997) The percentage of consonants correct (PCC) metric: extensions and reliability data. *J. Speech Lang. Hear. Res.,* **40,** 708-722.

26. Lewis, B.A. and Freebairn, L. (1993) A clinical tool for evaluating the familial basis of speech and language disorders. *Am. J. Speech Lang. Pathol.,* **2,** 38-43.

27. Lewis, B.A., Freebairn, L.A. and Taylor, H.G. (2000) Follow-up of children with early expressive phonology disorders. *J. Learn. Disabil.,* **33,** 433-444.

28. Robbins, J. and Klee, T. (1987) Clinical assessment of oropharyngeal motor development in young children. *J. Speech Hear. Disord.,* **52,** 271-277.

29. Fletcher, J. (1977) Fletcher Time-by-Count Test of Diadochokinetic Syllable Rate. C.C. Publications, Tigard, OR, USA.

30. Kamhi, A.G. and Catts, H.W. (1986) Toward an understanding of developmental language and reading disorders. *J. Speech Hear. Disord.,* **51,** 337-347.

31. Catts, H.W. (1986) Speech production/phonological deficits in reading-disordered children. *J. Learn. Disabil.,* **19,** 504-508.

32. Denckla, M.B. and Rudel, R.G. (1976) Naming of object-drawings by dyslexic and other learning disabled children. *Brain. Lang.,* **3,** 1-15.

33. Wiig, E.H., Secord, W. and Semel, E. (1992) Clinical Evaluation of Language Fundamentals - Preschool (CELF-P). The Psychological Corporation, San Antonio, TX, USA.

34. Newcomer, P.L. and Hammill, D.D. (1997) Test of Language Development - Primary, Third Edition (TOLD-P:3). Pro-Ed, Austin, TX, USA.

35. Dunn, L.M. and Dunn, L.M. (1997*) Peabody Picture Vocabulary Test - Third Edition (PPVT-III).* American Guidance Services, Circle Pines, MN, USA.

36. Gardner, M.F. (1990) Expressive One Word Picture Vocabulary Test - Revised (EOWPVT-R). Academic Therapy Publications, Novato, CA, USA.

37. Woodcock, R. (1987) Woodcock Reading Mastery Test-Revised (WRMT-R). American Guidance Services, Circle Pines, MN, USA.

38. Wechsler, D. (1992) Wechsler Individual Achievement Test (WIAT). The Psychological Corporation, San Antonio, TX, USA.

39. Larsen, S.C. and Hammill, D.D. (1994) Test of Written Spelling - Third Edition (TWS-3). Pro-Ed., Austin, TX, USA.

40. Hammill, D.D. and Larsen, S.C. (1988) Test of Written Language - 2nd Edition (TOWL-2). Pro-Ed., Austin, TX, USA.

41. Wechsler, D. (1989) Wechsler Preschool and Primary Scale of Intelligence - Revised (WPPSI-R). The Psychological Corporation, San Antonio, TX, USA.

42. Wechsler, D. (1991) Wechsler Intelligence Scale for Children - Third Edition (WISC-III). The Psychological Corporation, San Antonio, TX, USA.

43. Gardner, M.F. (1996) Test of Auditory-Perceptual Skills –Revised. Psychological and Educational Publications INC., Hydesville, California, USA.

44. Newcomer, P.L. and Hammill, D.D. (1988) Test of Language Development - Primary, Second Edition (TOLD-P:2). Pro-Ed, Austin, TX, USA.

45. Catts, H.W., Adlof, S.M., Hogan, T.P. and Weismer, S.E. (2005) Are specific language impairment and dyslexia distinct disorders? *J. Speech Lang. Hear. Res.,* **48,** 1378-1396.

46. Dollaghan, C. and Campbell, T.F. (1998) Nonword repetition and child language impairment. *J. Speech Lang. Hear. Res.,* **41,** 1136-1146.

47. Wallace, G. and Hammill, D. (1994) Comprehensive Receptive and Expressive Vocabulary Test (CREVT). Pro-Ed., Austin, TX, USA.

48. Dunn, L.M. (1981) Peabody Picture Vocabulary Test - Revised (PPVT-R). American Guidance Services, Circle Pines, MN, USA.
